# Situational analysis of nutritional status among 1899 children presenting with cleft lip and/or palate in Indonesia

**DOI:** 10.7189/jogh.13.04127

**Published:** 2023-10-20

**Authors:** Anggun Rafisa, Erli Sarilita, Barbara Delage, Ronald G Munger, Peter A Mossey

**Affiliations:** 1 Department of Oral Biology, Faculty of Dentistry, Universitas Padjadjaran, Jatinangor, West Java, Indonesia; 2 Smile Train, New York City, New York, USA; 3 Centre for Epidemiologic Studies, Utah State University, Logan, Utah, USA; 4 Division of Oral Health Sciences and WHO Collaborating Centre for Oral Health & Craniofacial Anomalies, University of Dundee, Dundee, Scotland, UK

## Abstract

**Background:**

Given the increased risk of malnutrition in children with cleft lip and/or palate (CLP), determining their nutritional status is critical for preventing adverse surgical risks. However, no such disaggregated, national-level data are available in Indonesia. We aimed to determine the nutritional status of patients with clefts in Indonesia and to identify problems and solutions for malnutrition cases within the population.

**Methods:**

In this cross-sectional study, we considered records of individuals who underwent primary surgery for CLP in Smile Train-sponsored facilities in Indonesia between 1 January 2016 and 31 December 2021 (n = 18 480). We only included children under the age of five with an evaluation date prior to admission date and excluded subjects with invalid data values. We classified their nutritional status by z-scores according to the World Health Organization Child Growth Standard (2006). Malnutrition cases cover four indicators – stunting, wasting, underweight, and overweight. We compared the prevalence for malnutrition cases in children under the age of five using national health survey data.

**Results:**

We included 1899 records following data validation. The national prevalence of stunting (24.4%), wasting (12.5%), and overweight cases (12.9%) was high, while underweight cases (6.8%) were comparatively low. Statistical analyses showed significant differences in nutritional status based on length/height-for-age between girls and boys aged 0-5 months (*P* = 0.008) and 48-60 months (*P* = 0.001), and based on body mass index-for-age (*P* = 0.000) between girls and boys aged 0-5 months. Girls in different age groups exhibited a statistically significant difference in nutritional status based on length/height-for-age (*P* = 0.002) and weight-for-age (*P* = 0.017). Concurrent stunting and overweight were the most common forms of concurrent malnutrition (8.7%). We found a significant difference in the prevalence of underweight (*P* = 0.001) and overweight (*P* = 0.000) cases between children with CLP and those without CLP.

**Conclusions:**

Our findings highlight the importance of nutritional interventions for children with orofacial clefts in Indonesia, and the importance of age and gender in their design and implementation. Further investigation is necessary to explore the risks of overweight and concurrent malnutrition among this population.

Indonesia is a country with a fairly high prevalence of children with congenital disorders (59.3 per 1000 live births) compared to other countries in Southeast Asia [1]. Cleft lip and/or palate (CLP) or orofacial cleft ranked second in the sentinel survey of congenital anomalies conducted between September 2014 and March 2018 in 28 hospitals of 18 provinces in Indonesia (20.4% of 956 congenital birth cases) [2]. Children with CLP are at risk of malnutrition since they tend to have difficulty latching and/or sucking effectively [3,4]. Moreover, children under-five are generally vulnerable to malnutrition [5]. Aside from the child's difficulty to breastfeed, mothers of infants with CLP may experience high levels of stress that can interfere with breast milk production and breastfeeding, potentially contributing to infant malnutrition [6,7].

Poor nutritional status is associated with a higher rate of adverse surgical outcomes [8]. A surgical intervention increases metabolism as the body responds to surgical injuries; in turn, adequate nutritional intake should support the wound healing process [9]. Poor preoperative nutritional status may compromise the safety at surgery and the healing process. Skilled assistance to mothers with feeding their infants is critical for mitigating the risk of poor feeding and malnutrition and ensure timely and safe cleft surgery [10].

Prior studies have examined the nutritional status of children with CLP using different growth indicators to identify malnutrition. Some have found a higher prevalence of underweight among children with CLP compared to age-matched peers without CLP [6,11]. A study in children <18 years of age with CLP from lower-level socioeconomic backgrounds found a high prevalence of stunting in this population [12]. Another study found that the prevalence of underweight and stunting in children with CLP was clinically higher compared to peers without CLP, although the difference was not statistically significant [13]. Across studies and settings, values for the prevalence of wasting among children with CLP have ranged from 3.9% to 68.2% [11,14,15]. To our knowledge, no studies have specifically investigated the prevalence of overweight in children with CLP.

Despite numerous studies suggesting a key role for nutrition in the management of CLP, data on nutritional status in CLP patients are still very limited. We previously reported that only six of 620 published studies on orofacial clefts between 2010 and 2019 investigated feeding interventions in lower middle-income countries (LMICs) [16], with only one providing information on the nutritional status of patients with cleft in Indonesia (along with other countries) [6]. However, the study did not conduct sub-national analyses, making it difficult to recognise Indonesia's barriers in addressing nutritional challenges faced by children with cleft. Therefore, we aimed to determine the nutritional status of children at cleft treatment centres across the provinces of Indonesia, and to discuss barriers and opportunities.

## METHODS

### Study design and participants

In this cross-sectional study, we included records of individuals who underwent primary surgery for orofacial clefts in Smile Train-sponsored facilities in Indonesia based on information uploaded by local cleft care providers into Smile Train’s online medical database between 1 January 2016 and 31 December 2021 (n = 18480). We checked the data for duplicate identification numbers and selected the most recent evaluation dates (n = 17064). We only included children under the age of five with an evaluation date (when subjects’ weight and length/height were measured) prior to admission date (admission for surgery) (n = 14110) to assess the children's nutritional status prior to intervention.

### Statistical analyses

We analysed the children’s weight, length/height, age (chronological age in months at the time of anthropometric evaluation), sex, province of origin (province of residence), and province of primary surgery hospital (province of the hospital where the children received their primary cleft surgery).

We assessed nutritional status using growth indicators such as weight-for-age, length/height-for-age, and body mass index (BMI)-for-age, classified by z-scores according to the World Health Organization (WHO) 2006 Child Growth Standard [17]. In our study, malnutrition covered four indicators – stunting (severely and moderately stunted), wasting (severely and moderately wasted), underweight (severely and moderately underweight), and overweight (overweight and obese). We also examined concurrent malnutritional status, including concurrent stunting and overweight, concurrent stunting and underweight, concurrent wasting and underweight, and concurrent stunting, wasting, and underweight.

The variable data values of the children within the year and age range were validated using the WHO Anthro for personal computers 2010, version 3.2.2 (World Health Organization, Geneva, Switzerland) [18]. We excluded subjects with missing data values, which we defined as follows:

1. Non-availability of data values. Subjects with absent information on study variables were excluded.
2. Invalid data values – subjects with weight, length/height, or z-scores outside of the following range values:
  1. Weight: minimum 0.9 kilograms, maximum 58 kilograms
  2. Length/height: minimum 38 centimetres, maximum 150 centimetres
  3. Length/height-for-age z-score: minimum -6 standard deviations (SDs), maximum 6 SDs
  4. Weight-for-age z-score: minimum -6 SDs, maximum 5 SDs
  5. BMI-for-age z-score: minimum -5 SDs, maximum 5 SDs

We conducted all analyses in Microsoft Excel 2016, version 16.0 (Microsoft Corporation, Redmond, Washington, USA) and IBM SPSS Statistics, version 26.0 (IBM Corporation, Armonk, New York, USA). We reported the prevalence of malnutrition as a percentage and categorised it according to the prevalence threshold for each indicator of malnutrition [19,20]. We used non-parametric tests to compare two or more groups on a nominal or ordinal dependent variable among children with CLP. We performed a Kruskal-Wallis H test to assess whether statistically significant differences in nutritional status existed based on each growth indicator among sex and age groups and conducted further pairwise comparisons using Dunn's procedure with a Bonferroni correction for multiple comparisons. Furthermore, we analysed the association of the prevalence of each concurrent malnutrition between sex and age groups using a χ^2^ test if all cells had expected counts greater than five. We otherwise used a Fisher exact test to evaluate association between sex groups and Fisher-Freeman-Halton exact test to evaluate association between age groups.

We also examined the prevalence of all forms of malnutrition cases among children under-five according to the national health survey (Study on Nutritional Status in Indonesia/SSGI 2021) for comparison [21]. To reduce risk of bias, we only included provinces with more than 40 study subjects. We used boxplots to identify potential outliers within the groups and the Shapiro-Wilk test to assess if the normality of data distribution. We performed an independent *t*-test if we found no outliers and if the data were normally distributed; otherwise, we used the Mann-Whitney U test.

### Ethics

The Research Ethics Committee Universitas Padjadjaran (No.1026/UN6.KEP/ED/2021) approved the study prior to data collection. Smile Train partners gained consent from patients or their caregivers to collect and use their data for marketing, advertising, educational reasons, and for quality assessments and evaluations.

## RESULTS

Based on the data validation process for subjects within the age range (n = 14 110), 2293 subjects (16.3%) had valid weight and length/height information from which z-scores could be calculated. A further 394 subjects (17.2%) had invalid weight-for-age, length/height-for-age, and/or BMI-for-age z-scores, leaving 1899 subjects with complete and valid data values for inclusion (Figure S1 in the **Online Supplementary Document**).

**Figure 1:**
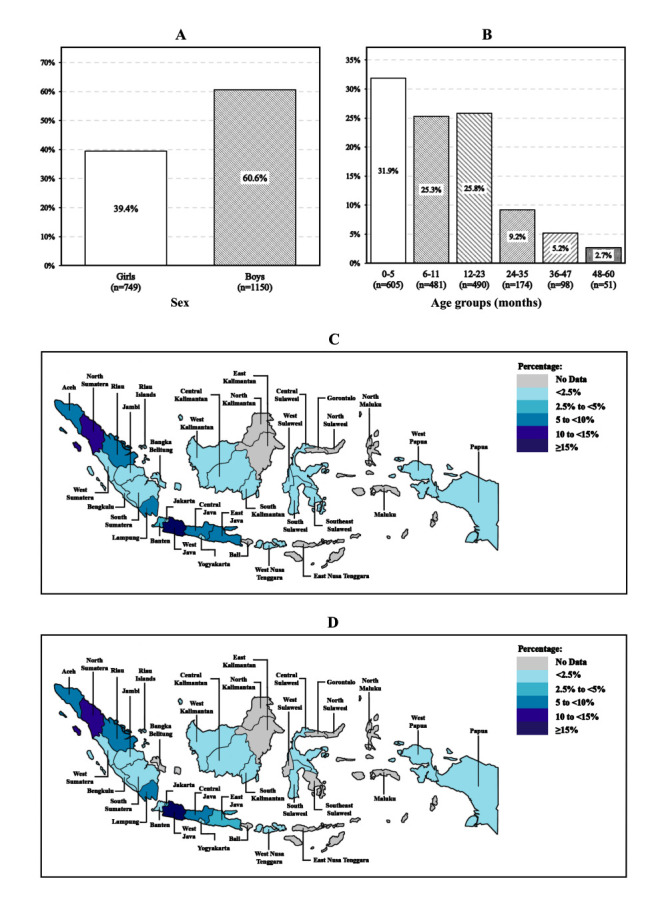
Frequency distribution of study subjects. **Panel A.** By sex. **Panel B.** By age groups. **Panel C.** By province of origin. **Panel D.** By province of primary surgery hospital.

Our sample consisted of more boys than girls, with an overall median age of nine months (interquartile range (IQR) = 5.0-19.0). The children with CLP came from 26 of Indonesia’s 34 provinces, predominantly from the western part of Indonesia, with most originating from the West Java Province (27.6%). Most children (91.4%) underwent primary surgery in their province of origin (**Figure 1**).

**Figure 2:**
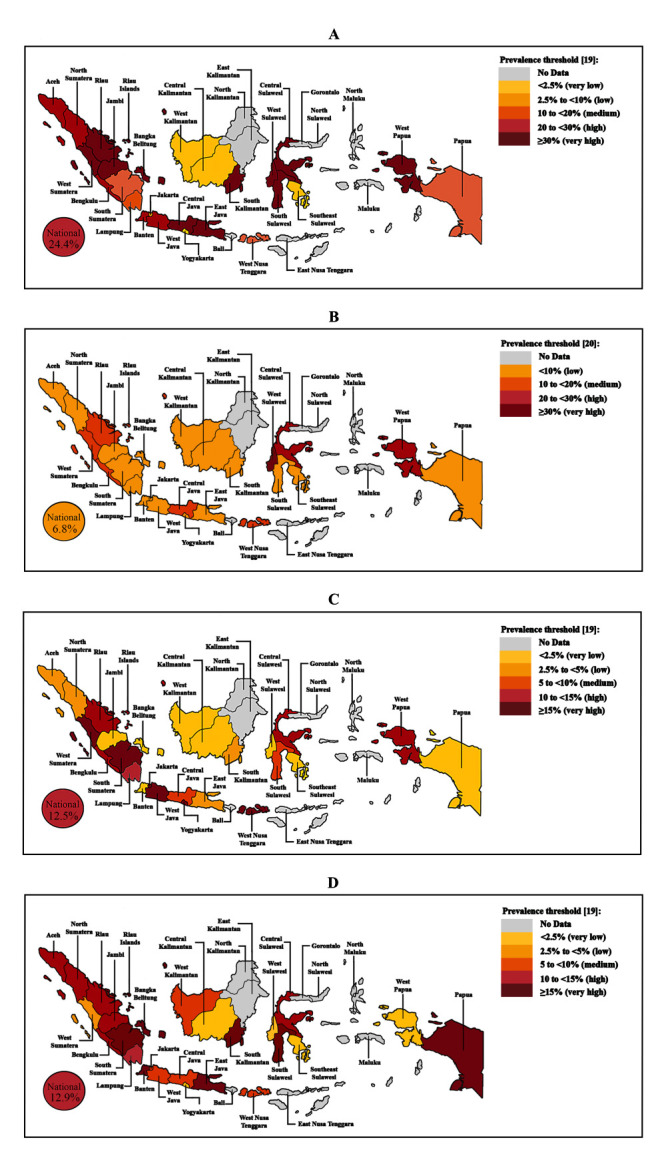
Geographical map of Indonesia presenting the prevalence of malnutrition cases among children under the age of five with CLP by province of origin. **Panel A.** Stunting cases. **Panel B.** Underweight cases. **Panel C.** Wasting cases. **Panel D.** Overweight cases.

Referring to the prevalence threshold for each form of malnutrition, we observed a high prevalence of stunting, wasting, and overweight among children with CLP and a low prevalence of underweight at the national level (see **Figure 2** for representation based on children’s province of origin and Table S1 in the **Online Supplementary Document** for details on prevalence of nutritional status among children with CLP).

**Figure 3:**
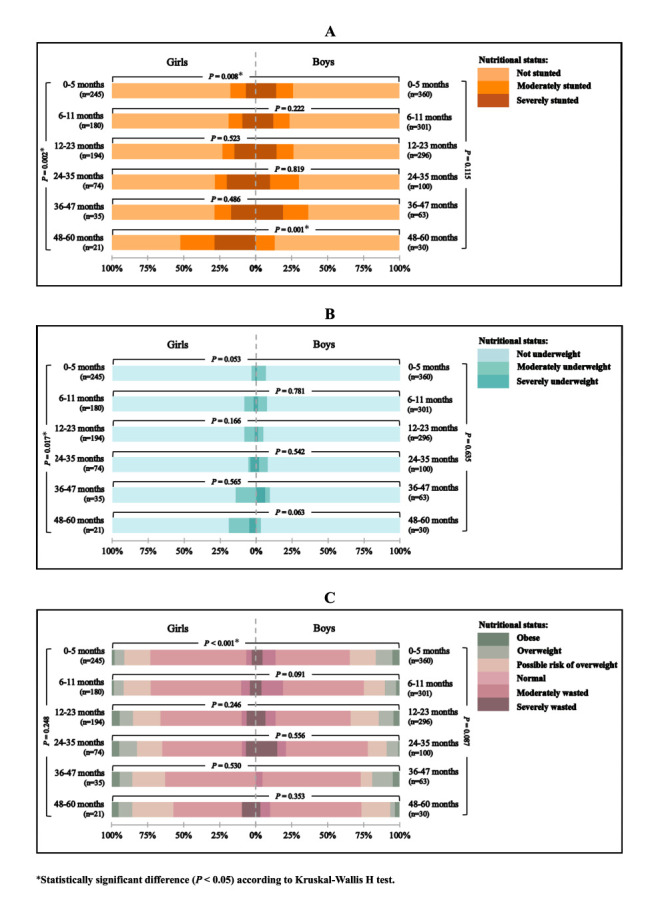
Comparison of nutritional status among children under the age of five with CLP between sex and age groups based on three growth indicators **Panel A.** Length/height-for-age. **Panel B.** Weight-for-age. **Panel C.** BMI-for-age. The left-hand bars represent the prevalence in girls and the right-hand bars represent the prevalence in boys. The horizontal brackets indicate the *P*-value of the statistical analysis between the sex category within the same age group, while the vertical brackets indicate the *P*-value of the statistical analysis between age groups within the same sex category.

We observed a statistically significant difference in nutritional status based on length/height-for-age (*P* = 0.008) and BMI-for-age (*P* < 0.001) between girls and boys aged 0-5 months and a significant difference was noted in nutritional status based on length/height-for-age between girls and boys aged 48-60 months (*P* = 0.001) (**Figure 3**). Furthermore, we found a statistically significant difference in nutritional status based on length/height-for-age (*P* = 0.002) and weight-for-age (*P* = 0.017) between different age groups in girls. The post hoc analysis, considering statistical significance at a *P* < 0.0083 level, showed significant differences in nutritional status among girls based on length/height-for-age between the 48-60 months and 12-23 months (*P* = 0.003), 48-60 months and 6-11 (*P* < 0.001), and 48-60 months and 0-5 months (*P* < 0.001) age groups. Additionally, we observed further statistically significant differences in nutritional status among girls based on weight-for-age between the 48-60 months and 0-5 months (*P* = 0.006) age groups.

**Figure 4:**
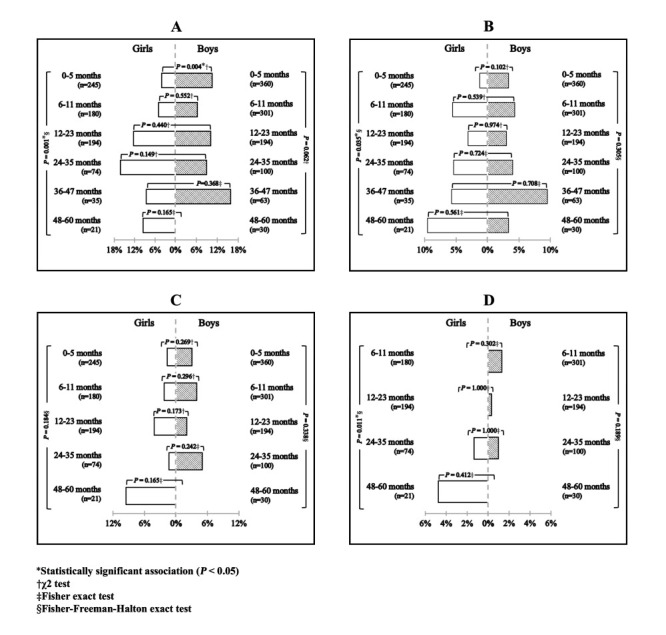
Comparison of the prevalence of concurrent malnutrition among children under-five with CLP between sex and age groups. **Panel A.** Concurrent stunting and overweight. **Panel B.** Concurrent stunting and underweight. **Panel C.** Concurrent wasting and underweight. **Panel D.** Concurrent stunting, wasting, and underweight. The left-hand bars represent the prevalence in girls and the right-hand bars represent the prevalence in boys. The horizontal brackets indicate the *P*-value of the statistical analysis between the sex category within the same age group, while the vertical brackets indicate the *P*-value of the statistical analysis between age groups within the same sex category. Age groups with no subjects in both sex categories is excluded from the graphical representation.

Concurrent stunting and overweight were the most common forms of concurrent malnutrition (8.7%). We found a statistically significant association in the prevalence of concurrent stunting and overweight between girls and boys aged 0-5 months (*P* = 0.003) and a statistically significant association between the age groups in the prevalence of concurrent stunting and overweight (*P* = 0.001), concurrent stunting and underweight (*P* = 0.035), and concurrent stunting, wasting, and underweight (*P* = 0.011) among girls (**Figure 4** and Table S2 in the **Online Supplementary Document**).

**Figure 5:**
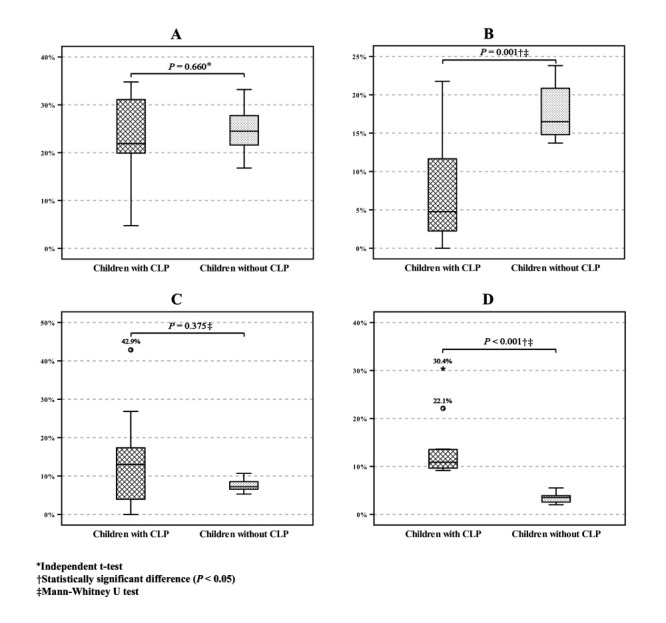
Comparison of the prevalence of malnutrition cases between children under the age of five with CLP and those without CLP in 11 provinces in Indonesia. **Panel A.** Stunting cases. **Panel B.** Underweight cases. **Panel C.** Wasting cases. **Panel D.** Overweight cases. The horizontal brackets indicate the *P*-value of the statistical analysis between the two groups. CLP – cleft lip and/or palate.

Regarding the comparison of prevalence of malnutrition cases among children under five with CLP and in the national data [19] (**Figure 5**), we only included 11 provinces (Aceh, North Sumatera, Riau, Lampung, Jakarta, West Java, Central Java, East Java, Banten, West Nusa Tenggara, and Central Sulawesi) due to an absence of the minimum size of study subjects in the remaining province. There was a statistically significant difference in the prevalence of underweight (*P* = 0.001) and overweight (*P* < 0.001) between children under-five with CLP and without CLP.

## DISCUSSION

Nutritional interventions for children with orofacial clefts have received little attention in Indonesia, where there is no available national disaggregated data on the feeding and nutritional needs of this population. Our analysis of anthropometric data collected prior to cleft surgeries in Indonesian health facilities over a five-year-period showed a high prevalence of stunting, wasting, and overweight in children with CLP and highlighted the urgent need for integrating nutrition care in the management of children with CLP.

We found that stunting cases, corresponding to chronic malnutrition, had the highest prevalence rate (24.4%) compared to other forms of malnutrition on a national scale. The rate is similar when compared to peers without clefts (24.4%) in SSGI 2021 [21]. Furthermore, we found no statistically difference in the prevalence of stunting between children with CLP and those without CLP across eleven provinces in Indonesia. Similarly, two case-control studies conducted in India [13] and Nigeria [22] (countries with a high prevalence of stunting in the general population) found no differences between children with cleft and without cleft.

Moreover, when examining the study subjects across different sex and age groups, we found a statistically significant difference in nutritional status based on length-for-age between boys and girls among the youngest age group (0-5 months), and a higher prevalence of stunting cases in boys than in girls within this particular group of children. Several studies have provided evidence supporting the notion that boys are at a higher risk of stunting [23,24], and this vulnerability can manifest as early as during the prenatal period [25]. This increased risk is attributed to their increased susceptibility to infections, particularly prevalent in lower socioeconomic families [25,26]. Nevertheless, a substantial increase in the number of stunting cases among girls was observed at the age of 48-60 months, leading to a significant difference in stunting prevalence between girls and boys. These disparities may be attributed to gendered cultural norms that influence feeding patterns, potentially resulting in higher stunting rates in girls. For instance, Ng et al. [27] discovered that the proportion of boys in Indonesia with adequate meal frequency was higher than girls. Similarly, another study analysing the 2005-2006 National Family Health Survey (NFHS-3) in India found that girls were breastfed for a shorter duration than boys [28]. Other factors such as parental diet, family socioeconomic level, and peer influences could also explain these findings [29]. Future research should investigate the association of these factors with sex differences in the prevalence of stunting among children with CLP to provide a deeper understanding and aid in formulating targeted interventions to address the underlying causes of disparities.

The national prevalence of underweight (6.8%) among children with CLP in the current study is considerably lower than peers without clefts in SSGI 2021 (17.0%). We found a significant difference in underweight prevalence between children with and without CLP, in contrast with findings of Babalola et al. [22]. The development of various techniques and tools designed to aid in feeding children with CLP [30], the availability of diverse professional support services for feeding consultations, and the increased awareness, effort, and success of mothers in nurturing and feeding these children [31-33] may be related to this observed low figure of underweight prevalence among children with CLP.

Although we observed a higher prevalence of underweight cases in boys than in girls, we found no significant variation in nutritional status based on weight-for-age between boys and girls within the same age group. However, we discovered a significant difference of nutritional status in this indicator between girls aged 0-5 months and those aged 48-60 months, with the higher prevalence of underweight was observed in the latter age group. Delage et al. [6] reported a higher prevalence of underweight among children with CLP aged 48-60 months compared to children aged 0-5 months, with a difference of 6.7%. Notably, children included in this study underwent anthropometric evaluations before surgery to assess their nutritional status prior to surgical intervention, which indicates that those children aged 48-60 months had been living with CLP for a longer duration compared to children aged 0-5 months. Lazarus et al [34] discovered that children who underwent cleft repair surgery after one year of age had a 1.5 times higher likelihood of becoming underweight than those who underwent it at a younger age. Delay in cleft repair surgery leads to prolonged feeding difficulties in children, particularly in children with cleft palate [35].

We identified a considerably higher prevalence of wasting (12.5%) and overweight (12.9%) among children with CLP at the national level compared to their peers in the national health survey (wasting: 7.1%, overweight: 3.8%) [21]. Notably, the comparison between children with and without CLP indicates a significant difference in the prevalence of overweight, while no significant difference is found for wasting, corroborating findings from previous studies [22].

This study does not show any significant differences in the nutritional status based on BMI-for-age between age groups in either girls or boys with CLP. Although boys exhibit a higher prevalence of wasting and overweight compared to girls, the significant difference is only evident between boys and girls aged 0-5 months. Interestingly, the prevalence of overweight cases in the youngest age group of boys is twice that of girls. This may be attributed to the higher prevalence of stunting in boys within this age group, as stunted children are more susceptible to becoming overweight [36].

Since children may suffer from multiple forms of malnutrition, we also examined the prevalence of concurrent malnutrition cases among children with CLP. We identified concurrent stunting and overweight as the most common form of concurrent malnutrition, affecting 8.7% of the study subjects, exceeding those observed among children under the age of five in Indonesia (5.6%) [37]. Concerns regarding overweight have arisen with the discovery of high prevalence of stunting among children with CLP, supported by several studies indicating a potential link between stunting and overweight, resulting in a risk of double burden of malnutrition [36,38,39]. Children with stunting may experience an increase in BMI when their food intake increases or becomes excessive, as weight gain tends to outpace height catch-up [40]. Moreover, children with CLP may have higher susceptibility on becoming overweight when they already stunted at birth. Previous studies confirmed that maternal nutritional status, such as overweight and micronutrient deficiencies are risk factors for having a child with clefts and that obese mothers tend to have obese babies [41-43]. It seems important to further study the link of nutritional status of mothers and the risk of overweight among babies with CLP.

Currently, limited attention has been given to studies examining overweight among children with CLP, with previous research primarily concentrating on the risk of undernutrition due to the feeding challenges experienced by these children. However, the finding of lower prevalence of underweight among study subjects in our study raises the question of whether current efforts to provide feeding care for children with clefts have indeed succeeded in reducing the prevalence of underweight but inadvertently led to the emergence of a new concern–a higher prevalence of overweight.

We also found that all other forms of concurrent malnutrition are also present among children with CLP. The analysis of the prevalence of concurrent malnutrition between sexes and age groups tends to align with the significance observed in single malnutrition forms. Multiple indicators of malnutrition are associated with an increased mortality risk [44]. Therefore, considering that children with CLP require surgical intervention to improve their quality of life, it becomes crucial to assess all indicators of malnutrition to avoid adverse surgical outcomes, including the most severe, mortality.

Apart from characteristics such as sex and age, we also collected geographical information on children with CLP. Our study did not rely on the national database registry, but rather on surgical records obtained from a humanitarian organisation specialising in cleft care, leading to significant differences in sample sizes across various provinces. We provided an overview of the distribution of malnutrition cases based on the children's province of origin as a reference for further research in the form of a geographical map (**Figure 2**). However, this map should be interpreted with caution and in reference to the distribution of subjects (**Figure 1**, panel C), as the high prevalence of malnutrition cases in certain provinces may be attributed to the low sample size.

In view of these geographic characteristics, most children with CLP underwent their primary surgery in the same province as their origin, suggesting that the presence of health care providers in the residency increases the likelihood of receiving CLP treatments. This finding is supported by a three-year survey study conducted by Massenburg et al. [45] in intercontinental low- and middle-income countries (LMICs), where patient travel costs were identified as the most commonly reported barrier to accessing CLP treatment.

Despite the concerns raised by this study regarding the prevalence of malnutrition among children with CLP, there is likely a noteworthy proportion of children who do not experience malnutrition according to various growth indicators (Table S1 in the **Online Supplementary Document**). Specifically, more than half (58.7%) of the children were not affected by any form of malnutrition. There has been a considerable decline in the prevalence of stunting cases among children under the age of five over the past decades globally, but also in Indonesia specifically [46]. We hope that a similar positive trend will occur for other forms of malnutrition, including in children with CLP, thereby contributing to the achievement of the second goal of the Sustainable Development Goals (SDGs) – zero hunger [47].

### Limitations

We did not assess socioeconomic status, which can significantly influence the nutritional status of children. Moreover, our subjects were patients presenting for primary surgery in Smile Train-sponsored facilities, meaning we did not include children who were not identified or those operated on in other facilities, potentially underestimating the true extent of the health care challenge. Moreover, subtle methodological differences in data collection in comparison to the control sample from national data may potentially limit our ability to directly compare the results. Likewise, more than 1% from the initial data sets had invalid z-scores, raising doubts about the quality of the data with valid z-scores. As we were unable to control the standardisation of anthropometric measurement and data input by local cleft care providers in the various facilities, we cannot exclude the possibility that z-scores value may be biased by incorrect dates of birth and anthropometric measurements.

## CONCLUSIONS

Our study highlights the importance of nutritional intervention for children with orofacial clefts, particularly in Indonesia, and the relevance of age and in designing and implementing these interventions. Further research should explore the risks of both overweight and concurrent malnutrition in these population, including a thorough examination of factors that influence the nutritional status of children, including genetics (maternal nutrition and family health behaviour), economics (income, food availability, and security), physical (access to food, maternal knowledge, and education), social (culture and diet pattern), and health services (access and use of health facilities).

## Additional material


Online Supplementary Document
