# Supplementary material for: Situational analysis of nutritional status among 1899 children presenting with cleft lip and/or palate in Indonesia
Source: J Glob Health. 2023 Oct 20;13:04127. doi: 10.7189/jogh.13.04127 (PMC10586796; doi:10.7189/jogh.13.04127)
Supplement: Online Supplementary Document [file jogh-13-04127-s001.pdf]

Figure S1. Flow diagram of study participants

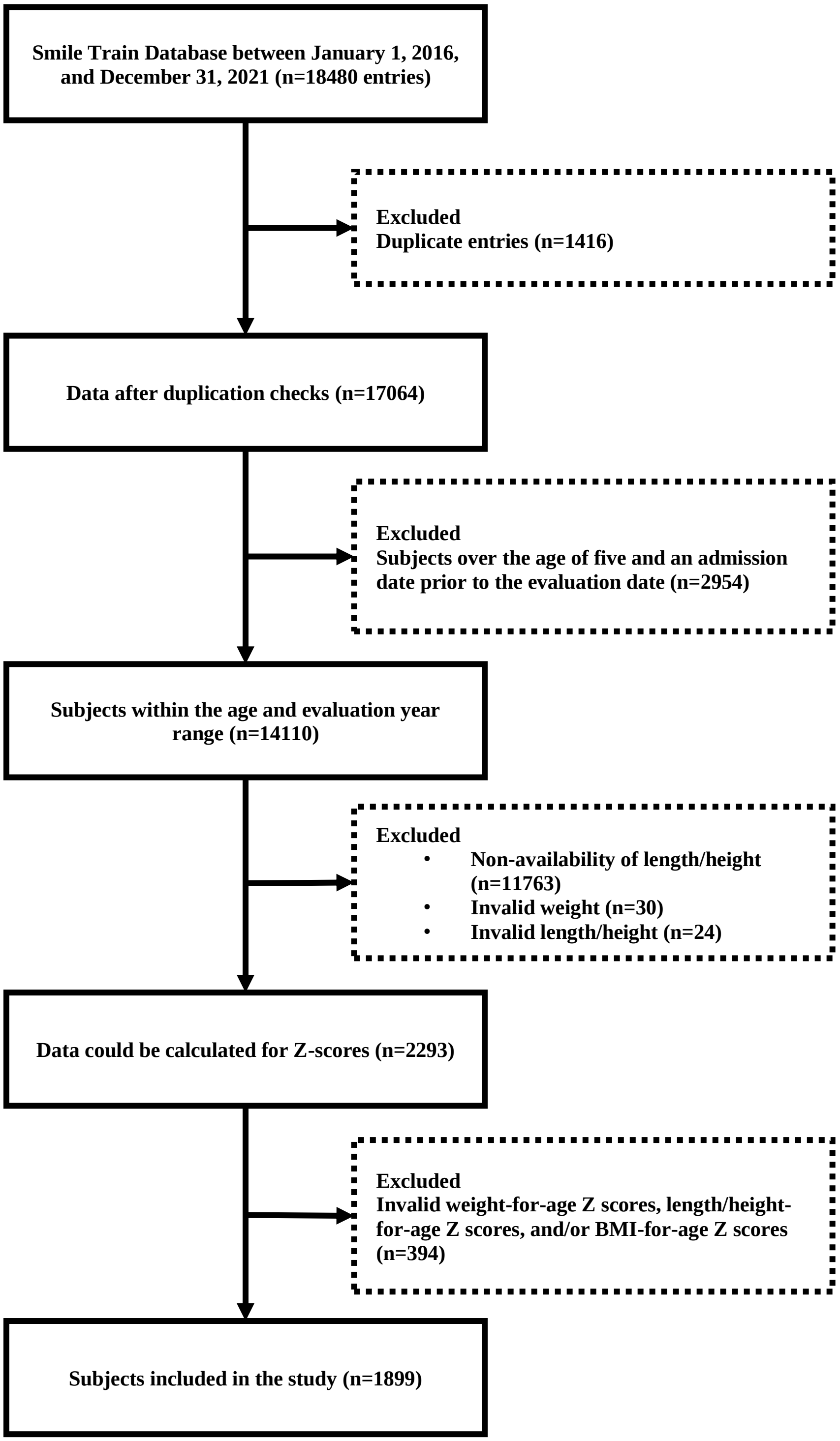

Table S1. Prevalence of nutritional status among children under the age of five with CLP

Growth Indicators	**Length/height-for-age**						**Weight-for-age**						**BMI-for-age**																	
	Severely stunted		Moderately Stunted		Not Stunted		Severely Underweight		Moderately Underweight		Not Underweight		Severely wasted		Moderately Wasted		Normal		Possible risk of overweight		Overweight		Obese	
	Count	Percent (95% CI)	Count	Percent (95% CI)	Count	Percent (95% CI)	Count	Percent (95% CI)	Count	Percent (95% CI)	Count	Percent (95% CI)	Count	Percent (95% CI)	Count	Percent (95% CI)	Count	Percent (95% CI)	Count	Percent (95% CI)	Count	Percent (95% CI)	Count	Percent (95% CI)
**Sex**																								
Girls (n=749)	90	12.0 (9.7-14.3)	74	9.9 (7.7-12.0)	585	78.1 (75.1-81.1)	11	1.5 (0.6-2.3)	41	5.5 (3.8-7.1)	697	93.1 (91.2-94.9)	33	4.4 (2.9-5.9)	29	3.9 (2.5-5.3)	458	61.1 (57.7-64.6)	142	19.0 (16.2-21.8)	61	8.1 (6.2-10.1)	26	3.5 (2.2-4.8)
Boys (n=1150)	154	13.4 (11.4-15.4)	146	12.7 (10.8-14.6)	850	73.9 (71.4-76.5)	16	1.4 (0.7-2.1)	62	5.4 (4.1-6.7)	1072	93.2 (91.8-94.7)	65	5.7 (4.3-7.0)	110	9.6 (7.9-11.3)	627	54.5 (51.6-57.4)	191	16.6 (14.5-18.8)	113	9.8 (8.1-11.5)	44	3.8 (2.7-4.9)
**Age groups (months)**																								
0-5 (n=605)	69	11.4 (8.9-13.9)	68	11.2 (8.7-13.8)	468	77.4 (74.0-80.7)	4	0.7 (0.0-1.3)	29	4.8 (3.1-6.5)	572	94.5 (92.7-96.4)	24	4.0 (2.4-5.5)	42	6.9 (4.9-9.0)	348	57.5 (53.6-61.5)	109	18.0 (15.0-21.1)	59	9.8 (7.4-12.1)	23	3.8 (2.3-5.3)
6-11 (n=481)	54	11.2 (8.4-14.0)	51	10.6 (7.9-13.4)	376	78.2 (74.5-81.9)	7	1.5 (0.4-2.5)	31	6.4 (4.3-8.6)	443	92.1 (89.7-94.5)	19	4.0 (2.2-5.7)	56	11.6 (8.8-14.5)	282	58.6 (54.2-63.0)	78	16.2 (12.9-19.5)	35	7.3 (5.0-9.6)	11	2.3 (1.0-3.6)
12-23 (n=490)	72	14.7 (11.6-17.8)	51	10.4 (7.7-13.1)	367	74.9 (71.1-78.7)	6	1.2 (0.3-2.2)	25	5.1 (3.2-7.1)	459	93.7 (91.5-95.8)	32	6.5 (4.3-8.7)	28	5.7 (3.7-7.8)	263	53.7 (49.3-58.1)	95	19.4 (15.9-22.9)	48	9.8 (7.2-12.4)	24	4.9 (3.0-6.8)
24-35 (n=174)	25	14.4 (9.2-19.6)	26	14.9 (9.6-20.2)	123	70.7 (63.9-77.5)	5	2.9 (0.4-5.4)	7	4.0 (1.1-6.9)	162	93.1 (89.3-96.9)	20	11.5 (6.8-16.2)	8	4.6 (1.5-7.7)	98	56.3 (49.0-63.7)	26	14.9 (9.6-20.2)	17	9.8 (5.4-14.2)	5	2.9 (0.4-5.4)
36-47 (n=98)	18	18.4 (10.7-26.0)	15	15.3 (8.2-22.4)	65	66.3 (57.0-75.7)	4	4.1 (0.2-8.0)	7	7.1 (2.0-12.2)	87	88.8 (82.5-95.0)	0	0.0	3	3.1 (0.0-6.5)	65	66.3 (57.0-75.7)	13	13.3 (6.5-20.0)	12	12.2 (5.8-18.7)	5	5.1 (0.7-9.5)

48-60 (n=51)	6	11.8 (2.9-20.6)	9	17.6 (7.2-28.1)	36	70.6 (58.1-83.1)	1	2.0 (0.0-5.8)	4	7.8 (0.5-15.2)	46	90.2 (82.0-98.4)	3	5.9 (0.0-12.3)	2	3.9 (0.0-9.2)	29	56.9 (43.3-70.5)	12	23.5 (11.9-35.2)	3	5.9 (0.0-12.3)	2	3.9 (0.0-9.2)
**Province of Origin**																								
Aceh (n=169)	21	12.4 (7.5-17.4)	16	9.5 (5.1-13.9)	132	78.1 (71.9-84.3)	0	0.0 (0.0-0.0)	4	2.4 (0.1-4.7)	165	97.6 (95.3-99.9)	1	0.6 (0.0-1.7)	7	4.1 (1.1-7.1)	100	59.2 (51.8-66.6)	38	22.5 (16.2-28.8)	12	7.1 (3.2-11.0)	11	6.5 (2.8-10.2)
North Sumatera (n=256)	23	9.0 (5.5-12.5)	29	11.3 (7.4-15.2)	204	79.7 (74.8-84.6)	1	0.4 0.0-1.2)	3	1.2 (0.0-2.5)	252	98.4 (96.9-100.0)	2	0.8 (0.0-1.9)	5	2.0 (0.3-3.6)	152	59.4 (53.4-65.4)	66	25.8 (20.4-31.1)	28	10.9 (7.1-14.8)	3	1.2 (0.0-2.5)
West Sumatera (n=23)	7	30.4 (11.6-49.2)	3	13.0 (0.0-26.8)	13	56.5 (36.3-76.8)	1	4.3 (0.0-12.7)	3	13.0 (0.0-26.8)	19	82.6 (67.1-98.1)	3	13.0 (0.0-26.8)	2	8.7 (0.0-20.2)	8	34.8 (15.3-54.2)	9	39.1 (19.2-59.1)	0	0.0	1	4.3 (0.0-12.7)
Riau (n=171)	31	18.1 (12.4-23.9)	24	14.0 (8.8-19.2)	116	67.8 (60.8-74.8)	5	2.9 (0.4-5.4)	20	11.7 (6.9-16.5)	146	85.4 (80.1-90.7)	16	9.4 (5.0-13.7)	7	4.1 (1.1-7.1)	89	52.0 (44.6-59.5)	36	21.1 (14.9-27.2)	13	7.6 (3.6-11.6)	10	5.8 (2.3-9.4)
Jambi (n=10)	1	10.0 (0.0-28.6)	3	30.0 (1.6-58.4)	6	60.0 (29.6-90.4)	0	0.0	0	0.0	10	100.0	0	0.0	0	0.0	7	70.0 (41.6-98.4)	2	20.0 (0.0-44.8)	1	10.0 (0.0-28.6)	0	0.0
South Sumatera (n=33)	3	9.1 (0.0-18.9)	3	9.1 (0.0-18.9)	27	81.8 (68.7-95.0)	0	0.0	1	3.0 (0.0-8.9)	32	97.0 (91.1-100.0)	2	6.1 (0.0-14.2)	3	9.1 (0.0-18.9)	19	57.6 (40.7-74.4)	4	12.1 (1.0-23.3)	5	15.2 (2.9-27.4)	0	0.0
Bengkulu (n=22)	4	18.2 (2.1-34.3)	1	4.5 (0.0-13.2)	17	77.3 (59.8-94.8)	0	0.0	3	13.6 (0.0-28.0)	19	86.4 (72.0-100.0)	1	4.5 (0.0-13.2)	2	9.1 (0.0-21.1)	10	45.5 (24.6-66.3)	6	27.3 (8.7-45.9)	3	13.6 (0.0-28.0)	0	0.0
Bangka Belitung (n=1)	1	100.0	0	0.0	0	0.0	0	0.0	0	0.0	1	100.0	0	0.0	0	0.0	0	0.0	0	0.0	0	0.0	1	100.0
Riau Islands (n=38)	4	10.5 (0.8-20.3)	10	26.3 (12.3-40.3)	24	63.2 (47.8-78.5)	1	2.6 (0.0-7.7)	4	10.5 (0.8-20.3)	33	86.8 (76.1-97.6)	1	2.6 (0.0-7.7)	3	7.9 (0.0-16.5)	17	44.7 (28.9-60.5)	11	28.9 (14.5-43.4)	5	13.2 (2.4-23.9)	1	2.6 (0.0-7.7)

Lampung (n=116)	10	8.6 (3.5-13.7)	7	6.0 (1.7-10.4)	99	85.3 (78.9-91.8)	1	0.9 (0.0-2.5)	2	1.7 (0.0-4.1)	113	97.4 (94.5-100.0)	8	6.9 (2.3-11.5)	8	6.9 (2.3-11.5)	60	51.7 (42.6-60.8)	28	24.1 (16.4-31.9)	8	6.9 (2.3-11.5)	4	3.4 (0.1-6.8)
Banten (n=56)	3	5.4 (0.0-11.3)	10	17.9 (7.8-27.9)	43	76.8 (65.7-87.8)	0	0.0	0	0.0	56	100.0	0	0.0	0	0.0	19	33.9 (21.5-46.3)	20	35.7 (23.2-48.3)	15	26.8 (15.2-38.4)	2	3.6 (0.0-8.4)
Jakarta (n=42)	1	2.4 (0.0-7.0)	1	2.4 (0.0-7.0)	40	95.2 (88.8-100.0)	0	0.0	2	4.8 (0.0-11.2)	40	95.2 (88.8-100.0)	5	11.9 (2.1-21.7)	13	31.0 (17.0-44.9)	16	38.1 (23.4-52.8)	4	9.5 (0.6-18.4)	3	7.1 (0.0-14.9)	1	2.4 (0.0-7.0)
West Java (n=525)	56	10.7 (8.0-13.3)	59	11.2 (8.5-13.9)	410	78.1 (74.6-81.6)	10	1.9 (0.7-3.1)	32	6.1 (4.0-8.1)	483	92.0 (89.7-94.3)	46	8.8 (6.3-11.2)	64	12.2 (9.4-15.0)	312	59.4 (55.2-63.6)	53	10.1 (7.5-12.7)	31	5.9 (3.9-7.9)	19	3.6 (2.0-5.2)
Central Java (n=153)	22	14.4 (8.8-19.9)	24	15.7 (9.9-21.4)	107	69.9 (62.7-77.2)	5	3.3 (0.5-6.1)	12	7.8 (3.6-12.1)	136	88.9 (83.9-93.9)	4	2.6 (0.1-5.1)	9	5.9 (2.2-9.6)	102	66.7 (59.2-74.1)	24	15.7 (9.9-21.4)	11	7.2 (3.1-11.3)	3	2.0 (0.0-4.2)
Yogyakarta (n=2)	0	0.0 (0.0-0.0)	0	0.0	2	100.0	0	0.0	0	0.0	2	100.0	0	0.0	1	50.0 (0.0-100.0)	1	50.0 (0.0-100.0)	0	0.0	0	0.0	0	0.0
East Java (n=95)	23	24.2 (15.6-32.8)	8	8.4 (2.8-14.0)	64	67.4 (57.9-76.8)	0	0.0	2	2.1 (0.0-5.0)	93	97.9 (95.0-100.0)	2	2.1 (0.0-5.0)	1	1.1 (0.0-3.1)	59	62.1 (52.3-71.9)	12	12.6 (6.0-19.3)	10	10.5 (4.4-16.7)	11	11.6(5.1-18.0)
West Nusa Tenggara (n=41)	3	7.3 (-0.7-15.3)	5	12.2 (2.2-22.2)	33	80.5 (68.4-92.6)	1	2.4 (0.0-7.2)	4	9.8 (0.7-18.8)	36	87.8 (77.8-97.8)	3	7.3 (0.0-15.3)	8	19.5 (7.4-31.6)	21	51.2 (35.9-66.5)	5	12.2 (2.2-22.2)	4	9.8 (0.7-18.8)	0	0.0
West Kalimantan (n=14)	0	0.0	0	0.0	14	100.0	0	0.0	0	0.0	14	100.0	0	0.0	0	0.0	11	78.6 (57.1-100.0)	2	14.3 (0.0-32.6)	1	7.1 (0.0-20.6)	0	0.0
Central Kalimantan (n=4)	0	0.0	0	0.0	4	100.0	0	0.0	0	0.0	4	100.0	0	0.0	0	0.0	4	100.0	0	0.0	0	0.0	0	0.0

South Kalimantan (n=27)	6	22.2 (6.5-37.9)	3	11.1 (0.0-23.0)	18	66.7 (48.9-84.4)	0	0.0	0	0.0	27	100.0	0	0.0	1	3.7 (0.0-10.8)	15	55.6 (36.8-74.3)	3	11.1 (0.0-23.0)	7	25.9 (9.4-42.5)	1	3.7 (0.0-10.8)
West Sulawesi (n=1)	1	100.0	0	0.0	0	0.0	0	0.0	1	100.0	0	0.0	0	0.0	0	0.0	0	0.0	1	100.0	0	0.0	0	0.0
South Sulawesi (n=35)	12	34.3 (18.6-50.0)	5	14.3 (2.7-25.9)	18	51.4 (34.9-68.0)	0	0.0	0	0.0	35	100.0	1	2.9 (0.0-8.4)	1	2.9 (0.0-8.4)	17	48.6 (32.0-65.1)	4	11.4 (0.9-22.0)	10	28.6 (13.6-43.5)	2	5.7 (0.0-13.4)
Central Sulawesi (n=46)	10	21.7 (9.8-33.7)	6	13.0 (3.3-22.8)	30	65.2 (51.5-79.0)	1	2.2 (0.0-6.4)	9	19.6 (8.1-31.0)	36	78.3 (66.3-90.2)	3	6.5 (0.0-13.7)	3	6.5 (0.0-13.7)	31	67.4 (53.8-80.9)	4	8.7 (0.6-16.8)	5	10.9 (1.9-19.9)	0	0.0
Southeast Sulawesi (n=1)	0	0.0	0	0.0	1	100.0	0	0.0	0	0.0	1	100.0	0	0.0	0	0.0	1	100.0	0	0.0	0	0.0	0	0.0
West Papua (n=7)	1	14.3 (0.0-40.2)	2	28.6 (0.0-62.0)	4	57.1 (20.5-93.8)	1	14.3 (0.0-40.2)	1	14.3 (0.0-40.2)	5	71.4 (38.0-100.0)	0	0.0	1	14.3 (0.0-40.2)	5	71.4 (38.0-100.0)	1	14.3 (0.00-40.2)	0	0.0	0	0.0
Papua (n=11)	1	9.1 (0.0-26.1)	1	9.1 (0.0-26.1)	9	81.8 (59.0-100.0)	0	0.0	0	0.0	11	100.0	0	0.0	0	0.0	8	72.7 (46.4-99.0)	1	9.1 (0.00-26.1)	2	18.2 (0.00-41.0)	0	0.0
**Total (n=1899)**	**244**	**12.8 (11.3-14.4)**	**220**	**11.6 (10.1-13.0)**	**1435**	**75.6 (73.6-77.5)**	**27**	**1.4 (0.9-2.0)**	**103**	**5.4 (4.4-6.4)**	**1769**	**93.2 (92.0-94.3)**	**98**	**5.2 (4.2-6.2)**	**139**	**7.3 (6.1-8.5)**	**1085**	**57.1 (54.9-59.4)**	**333**	**17.5 (15.8-19.2)**	**174**	**9.2 (7.9-10.5)**	**70**	**3.7 (2.8-4.5)**

CI – confidence interval

Table S2. Prevalence of concurrent malnutrition among children under the age of five with CLP

	Concurrent stunting and overweight		Concurrent stunting and underweight		Concurrent wasting and underweight		Concurrent stunting, wasting, and underweight	
	Count	Percent (95% CI)	Count	Percent (95% CI)	Count	Percent (95% CI)	Count	Percent (95% CI)
**Sex**								
Girls (n=749)	60	8.0 (6.1-10.0)	27	3.6 (2.3-4.9)	19	2.5 (1.4-3.7)	2	0.3 (0.0-0.6)
Boys (n=1150)	106	9.2 (7.5-10.9)	45	3.9 (2.8-5.0)	34	3.0 (2.0-3.9)	6	0.5 (0.1-0.9)
**Age groups (months)**								
0-5 (n=605)	48	7.9 (5.8-10.1)	15	2.5 (1.2-3.7)	15	2.5 (1.2-3.7)	0	0.0
6-11 (n=481)	28	5.8 (3.7-7.9)	23	4.8 (2.9-6.7)	16	3.3 (1.7-4.9)	4	0.8 (0.0-1.6)
12-23 (n=490)	54	11.0 (8.2-13.8)	15	3.1 (1.5-4.6)	14	2.9 (1.4-4.3)	1	0.2 (0.0-0.6)
24-35 (n=174)	21	12.1 (7.2-16.9)	8	4.6 (1.5-7.7)	6	3.4 (0.7-6.2)	2	1.1 (0.0-2.7)
36-47 (n=98)	13	13.3 (6.5-20.0)	8	8.2 (2.7-13.6)	0	0.0	0	0.0
48-60 (n=51)	2	3.9 (0.0-9.2)	3	5.9 (0.0-12.3)	2	3.9 (0.0-9.2)	1	2.0 (0.0-5.8)
**Province of Origin**								
Aceh (n=169)	18	10.7 (6.0-15.3)	3	1.8 (0.0-3.8)	0	0.0	0	0.0
North Sumatera (n=256)	24	9.4 (5.8-12.9)	2	0.8 (0.0-1.9)	2	0.8 (0.0-1.9)	0	0.0
West Sumatera (n=23)	1	4.3(-4.0-12.7)	2	8.7 (0.0-20.2)	2	8.7 (0.0-20.2)	1	4.3 (0.0-12.7)

Riau (n=171)	16	9.4 (5.0-13.7)	15	8.8 (4.5-13.0)	12	7.0 (3.2-10.8)	3	1.8 (0.0-3.7)
Jambi (n=10)	1	10.0 (0.0-28.6)	0	0.0	0	0.0	0	0.0
South Sumatera (n=33)	2	6.1 (0.0-14.2)	1	3.0 (0.0-8.9)	0	0.0	0	0.0
Bengkulu (n=22)	2	9.1 (0.0-21.1)	0	0.0	2	9.1 (0.0-21.1)	0	0.0
Bangka Belitung (n=1)	1	100.0	0	0.0	0	0.0	0	0.0
Riau Islands (n=38)	3	7.9 (0.0-16.5)	3	7.9 (0.0-16.5)	2	5.3 (0.0-12.4)	1	2.6 (0.0-7.7)
Lampung (n=116)	9	7.8 (2.9-12.6)	2	1.7 (0.0-4.1)	0	0.0	0	0.0
Banten (n=56)	9	16.1 (6.5-25.7)	0	0.0	0	0.0	0	0.0
Jakarta (n=42)	1	2.4 (0.0-7.0)	1	2.4 (0.0-7.0)	1	2.4 (0.0-7.0)	0	0.0
West Java (n=525)	29	5.5 (3.6-7.5)	24	4.6 (2.8-6.4)	17	3.2 (1.7-4.8)	2	0.4 (0.0-0.9)
Central Java (n=153)	8	5.2 (1.7-8.8)	9	5.9 (2.2-9.6)	5	3.3 (0.5-6.1)	0	0.0
Yogyakarta (n=2)	0	0.0	0	0.0	0	0.0	0	0.0
East Java (n=95)	17	17.9 (10.2-25.6)	0	0.0	2	2.1 (0.0-5.0)	0	0.0
West Nusa Tenggara (n=41)	3	7.3 (0.0-15.3)	1	2.4 (0.0-7.2)	4	9.8 (0.7-18.8)	0	0.0
West Kalimantan (n=14)	0	0.0	0	0.0	0	0.0	0	0.0
Central Kalimantan (n=4)	0	0.0	0	0.0	0	0.0	0	0.0

South Kalimantan (n=27)	5	18.5 (3.9-33.2)	0	0.0	0	0.0	0	0.0
West Sulawesi (n=1)	0	0.0	1	100.0	0	0.0	0	0.0
South Sulawesi (n=35)	11	31.4 (16.0-46.8)	0	0.0	0	0.0	0	0.0
Central Sulawesi (n=46)	5	10.9 (1.9-19.9)	6	13.0 (3.3-22.8)	3	6.5 (0.0-13.7)	0	0.0
Southeast Sulawesi (n=1)	0	0.0	0	0.0	0	0.0	0	0.0
West Papua (n=7)	0	0.0	2	28.6 (0.0-62.0)	1	14.3 (0.0-40.2)	1	14.3 (0.0-40.2)
Papua (n=11)	1	9.1 (0.0-26.1)	0	0.0	0	0.0	0	0.0
**Total (n=1899)**	**166**	**8.7 (7.5-10.0)**	**72**	**3.8 (2.9-4.7)**	**53**	**2.8 (2.1-3.5)**	**8**	**0.4 (0.1-0.7)**

CI – confidence interval
